# The influence of ergosterol on termites’ defenses against *Metarhizium* infections

**DOI:** 10.1007/s00114-026-02183-3

**Published:** 2026-10-12

**Authors:** Jacob H. Rosenbloom, Bhawana Luitel, Mark S. Bulmer

**Affiliations:** Department of Biological Sciences, Towson University, 4101 Science Complex, 8000 York Rd, Towson, MD 21252 USA

**Keywords:** Social immunity, Defensin, Termicin, Entomopathogen, Allogrooming

## Abstract

Termites respond to spores of pathogenic fungi that have adhered to the cuticle with brief bouts of rapid shaking, an alarm response that promotes allogrooming, which helps prevent fatal infections. The pathogen associated molecular pattern (PAMP) that elicits this proactive hygienic response is unknown. Ergosterol, an important component of the fungal cell membrane, has been identified as a potential PAMP in the subterranean termite *Coptotermes formosansus*. In this study, commercial or extracted ergosterol elicited a hygienic behavioral response in workers of the subterranean termite *Reticulitermes flavipes*. The ergosterol content of pathogenic spores of *Metarhizium* spp. were also found to vary among different strains and species of the fungus. However, there was a significant positive correlation between ergosterol content of different *Metarhizium* isolates and fatal infections caused by their spores (conidia). Alarm responses from challenges with conidia were not positively correlated with estimates of ergosterol content and instead were negatively correlated with ergosterol content although not significantly, which does not support the hypothesis that ergosterol is a critical recognition cue for the hygienic response. Termicin, an antifungal peptide in termite salivary secretions, was also most effective against the most lethal isolates, which had the highest ergosterol content. Ergosterol appears to influence spore membrane vulnerability to disruption by the antifungal peptide. Overall, our results indicate ergosterol influences both the vulnerability of *Metarhizium* conidia to termicin and the detection of an unidentified PAMP, which alerts termites to fungal contamination. This sets up a potential trade-off for the fungus between a shield for protection and signal for detection: reducing vulnerability to termicin by decreasing the content of cell membrane ergosterol results in an increase in a cell wall PAMP that termites can recognize and respond to with allogrooming. *Reticulitermes* spp. appear to have adaptively exploited this trade-off.

## Introduction

Fungi are among the most common insect pathogens, and many can directly penetrate the insect exoskeleton or cuticle (Lu and Leger 2016). An effective cuticular barrier is critical for survival especially when the internal innate immune system cannot cope with infection. After infecting the host *Metarhizium* spp., which are ubiquitous in soil, mask and modify their cell walls to evade detection by the innate immune system (Wang and St. Leger 2006; Wang et al. 2023; Zhang et al. 2024). Subterranean termites including *Reticulitermes* species have a relatively thin cuticle compared to many other insects that are commonly found in soil (Zukowski and Su 2020; Peeters et al. 2017). Secreted antimicrobial proteins (AMPs) appear to help termites limit their susceptibility to fatal infections by *Metarhizium* (Hamilton et al. 2011). Their exposure appears to have increased when termites transitioned from occupying a single piece of wood to foraging for food that required moving through soil. The selective pressure on β-1,3-glucanases, which help provide external protection by directly damaging the integrity of the fungal cell wall, intensified with this transition to foraging at multiple sites (Bulmer and Stefano 2022).

Social insects that move through the soil have to persistently contend with *Metarhizium* conidia. Elevated allogrooming in termites and ants appears to be critical for removal of contaminating conidia before they germinate and penetrate the insects’ cuticle (Chouvenc et al. 2009; Bulmer et al. 2019; Stock et al. 2023). When *Reticulitermes* workers encounter spores of *Metarhizium* they often respond with aggregation and allogrooming (Bulmer et al. 2019). Workers quickly remove spores (conidia) that have attached to their nestmates by cleaning each other’s cuticles with their mouthparts. Ingested conidia are effectively eliminated and β-1,3-glucanases and termicins that are produced by the salivary gland appear to be important antimycotics in this cleaning process (Lamberty et al. 2001; Hamilton and Bulmer 2012; Chouvenc et al. 2008, 2009; Bulmer et al. 2023). Moreover, termite workers sense the level of contamination on their nestmates and adaptively adjust their cleaning effort. Workers that have detected contamination will display alarm with brief bouts of rapid jerking movements of their whole body (Rosengaus et al. 1999). This alarm behavior appears to be a call for help that solicits allogrooming from nestmates (Bulmer et al. 2019, 2023).

It is unclear how social insects detect conidia that have adhered to the cuticle. Ergosterol is an important stabilizing component of the fungal cell membrane (Rodrigues 2018), which appears to influence the composition of the cell wall. Fungal cell wall integrity and rigidity are influenced by ergosterol content and research on antifungals that target ergosterol synthesis suggests that its reduction in the cell membrane is compensated by enhancing the integrity of the cell wall (Mesa-Arango et al. 2016; Ribeiro et al. 2022). Treatment of termite worker cuticle with ergosterol elicits increased allogrooming in *Coptotermes formosansus*, a rhinotermitid that is closely related to *Reticulitermes* (Chen et al. 2023). Ergosterol was also detected by GC-MS on the cuticular surface of *C. formosansus* workers that been challenged with *M. anisopliae.* In Argentine ants, a reduction in ergosterol concentration in *Metarhizium* appears to be associated with reduced allogrooming in response to a fungal challenge (Stock et al. 2023). Ergosterol may therefore act directly as a pathogen associated molecular pattern (PAMP) that elicits behavioral responses or indirectly by influencing other PAMPs in the cell membrane or wall.

In addition to a role as a PAMP for pathogen detection, ergosterol appears to influence the antimicrobial activity of antimycotics, including defensins, which disrupt the fungal cell membrane (Eliaš et al. 2024). There is a significant correlation between the antifungal activity of a termite defensin (termicin) against conidia from different strains and species of *Metarhizium* and the alarm behavior they induce in termites (Luitel et al. 2024). This correlation suggests that PAMPs that affect termite recognition of conidial contamination and the onset of elevated allogrooming also affect the vulnerability of conidia to the disruption of their cell membranes by termicin. The peptides’ antifungal activity appears to be most effective when conidia of *Metarhizium* isolates pose the most serious threat because they elicit a weak social immune response.

There has been a recent selective sweep for an advantageous variant of termicin in two sympatric species of *Reticulitermes* (*R. flavipes* and *R. virginicus*) in Maryland (Bulmer et al. 2010). Changes in termicin may be an adaptive response to changes in the properties of the cell membrane of a specific *Metarhizium* strain. Alternatively, the regional and local distribution of different fungal pathogen strains that slowly shift in their prevalence in the rhizosphere could expose termites to different PAMPs that require adaptive change in termite AMPs. Interestingly, *R. flavipes* and *R. virginicus* workers from the same locale differ in their social immune response to two commonly encountered species of *Metarhizium* (*M. brunneum* and *M. robertsii*), which suggests they have co-adapted to different strains or species of the fungus in their recent past (Bulmer et al. 2023). Highly pathogenic isolates that elicit a weak social immune response could therefore be responsible for selection of a termicin variant that is effective at targeting them.

In this study, we tested the hypotheses that ergosterol can elicit a behavioral response in *R. flavipes* workers and whether variation in ergosterol content in different isolates of *Metarhizium* affects both recognition of the fungus and the effectiveness of termicin.

## Methods

### Study organisms

Foraging groups of *R. flavipes* (> 1000) and the wood in which they were feeding were collected from secondary growth deciduous forest in Baltimore County, Maryland and stored in plastic containers under dark moist conditions at 25 °C. Foraging termites were collected from sites that were separated by at least 60 m, to help ensure that collections were from different colonies (Vargo and Husseneder 2009).

The *Metarhizium* species were isolated from soil samples usually next to active termite foraging sites (< 30 cm) using a baiting technique with *Tenebrio molitor* larvae (Denier and Bulmer 2015). In this study we used clonally propagated isolates: six different isolates of *Metarhizium robertsii* (Mr), five different isolates of *Metarhizium brunneum* (Mb) and two different isolates of *Metarhizium guizhouense* (Mg). These isolates were collected from Texas (*n* = 2; Mr), North Carolina (*n* = 2; Mr and Mg), Virginia (*n* = 1; Mb), Maryland (*n* = 6; 3 Mb, 2 Mr, 1 Mg) and Massachusetts (*n* = 2; Mr and Mb), USA.

*Metarhizium* species were identified with DNA sequence from the 5’ region of translation elongation factor 1-alpha (EFα1, Genbank accession numbers PZ946207 and PZ842080-PZ842091) using PCR primers EF1T and EF2T (Bischoff et al. 2009) and strains of the same species were differentiated with sequence from an ITS region (partial sequence of 18 S ribosomal RNA, the internal transcribed spacer 1, 5.8 S ribosomal RNA, internal transcribed spacer 2, and partial sequence of 28 S ribosomal RNA) using PCR primers PN3 and PN34 (Viaud et al. 2000), and an IGS region (partial sequence of the flanking ribosomal RNA intergenic spacer) using PCR primers Ma-IGSspR and Ma-IGSspF (Pantou et al. 2003).

### Termite survivorship and alarm

Groups of 12 *R. flavipes* workers were placed in Petri-dishes (47 mm diameter) lined with water moistened filter paper for 24 h to acclimate. The groups of 12 workers were then collectively exposed to 500 µl 10^6^ conidia ml^− 1^ of water or water alone for controls by brief immersion and agitation for ten seconds in 1.5 ml tubes (Bulmer et al. 2019). The conidia from each isolate were initially suspended in 0.1% Tween 80 to help break up spore clumps and subsequently diluted in sterile water to 10^6^ conidia ml^− 1^ for treating termites, so that Tween 80 did not interfere with spore adhesion to the cuticle. Soldiers were not included as they do not groom nestmates, represent 1–2% of the colony and are not consistently found in foraging groups. Immediately after fungal or control treatments, termites were transferred to Petri-dishes lined with moistened filter paper and were video recorded for 12 min. Mortality was subsequently monitored daily for 2 weeks, and dead individuals were removed for confirmation of infection by *Metarhizium* (green muscardine growth after 3–4 days), which was consistently above 95% for workers treated with conidia. Alarm, brief bouts of longitudinal oscillatory movement (LOM) lasting 2–7 s, was recorded for all workers during the first 12 min after fungal or control challenges with 3–4 replicates for each fungal isolate and 4 replicates for the control. The replicates for each isolate were composed of termites from a least two separate colonies. LOMs counted as separate events if the LOM completely stopped and then restarted. LOMs in the first 12 min are a convenient surrogate for measuring the intensification of allogrooming (Bulmer et al. 2019).

### Preference tests for ergosterol

Circular filter papers (Fisher Brand, cellulose filter pad for 47 mm diameter tissue culture Petri-dish) were cut into two halves. Each half was soaked with either 500 µl 1 mg/ml ergosterol (Sigma-Aldrich) in 100% ethanol, 500 µl 1 mg/ml cholesterol (Sigma-Aldrich) in 100% ethanol, or 500 µl 100% ethanol alone. The ethanol was allowed to evaporate from the filter papers overnight. Filter paper halves were then remoistened with 500 µl of sterile water. The treated and untreated filter paper halves were placed into Petri-dishes as follows: ergosterol-treated vs. ethanol-treated, cholesterol-treated vs. ethanol-treated, and ethanol-treated vs. ethanol-treated (control). Groups of 12 workers from two colonies (four replicates for each colony) were kept aside in separate Petri-dishes lined with water moistened filter paper for 24 h. Termites were then introduced into the Petri-dishes with paired filter paper halves, and video recorded for 12 min. The number of termites on each side of the filter paper was counted every minute and the frequency of LOMs and allogrooming bouts were recorded over 12 min. Allogrooming behavior, defined as mouth-to-body contact between two termites with movement of their mouthparts (palps), was recorded as separate events only if the termites broke contact and then initiated new contact (Bulmer et al. 2019).

### Ergosterol extracts

*Metarhizium* isolates (*n* = 13) were grown on potato dextrose agar plates (9 cm diameter Petri-dishes) supplemented with 50 µg/ml ampicillin until sporulation (2–3 weeks). The agar, mycelia and conidia from half a plate were cut into pieces and placed in a 50 ml polypropylene tube (Fisher Brand) with 20–25 ml 0.1% Tween 80 and shaken vigorously for 10 min. The resulting suspension was filtered through cheese cloth (XelNaga muslin) to separate conidia from agar pieces and mycelia. The filtrate was centrifuged in a 50 ml tube (21 °C, 3220 G, 10 min) and the supernatant was removed. The 50 ml tube and conidia pellet were weighed before suspending the pellet in 600 µl 25% alcoholic potassium hydroxide solution (2.5 g KOH and 3.5 ml sterile H_2_O made up to 10 ml with 100% ethanol). The conidia suspensions were vortexed vigorously for 1 min, transferred to 11 ml Pyrex glass tubes with phenolic screw caps (Corning™), and incubated in an 85 °C water bath for 1 h (Arthington-Skaggs et al. 1999). The 50 ml tube without the pellet was allowed to fully dry before reweighing to determine the wet weight of the pellet. The suspension was then allowed to cool before adding 200 µl sterile H_2_O and 600 µl *n-*heptane followed by vortex mixing for 3 min. The clear heptane layer was transferred to a clean Pyrex tube and stored at -20 °C for as long as 24 h. The absorbance of the saponified heptane extract was measured at 230 and 281.5 nm (Shimadzu UV-1800) as well as scanned between 220 and 300 nm in order to compare the extract profiles to spectrophotometric grade ergosterol (Sigma-Aldrich).

Non saponified methanol extracts were prepared by vortexing the conidia pellet, prepared as described above, in 2 ml of 120 mM sodium acetate (pH 5). One ml of the suspension was transferred to a 1.5 ml polypropylene tube and centrifuged at 11,000 G for 3 min. The pellet was resuspended in 100% methanol, homogenized in a glass mortar and pestle, and mixed on a benchtop shaker for 1 h in a 1.5 ml polypropylene tube. After shaking, the homogenate was centrifuged at 11,000 G for 10 min and the supernatant was stored at -20 °C for up to 24 h prior to analysis with the spectrophotometer as described above.

### Alarm response to ergosterol extracts

Methanol extracts of ergosterol were prepared as described above for 13 *Metarhizium* isolates. Cellulose filter papers (47 mm diameter) were soaked with 1 ml of methanol extract obtained from 50 mg of the wet conidia pellet, or methanol for a negative control and 300 µg/ml ergosterol (Sigma-Aldrich) for a positive control. The cellulose was completely dried for 24 h at 20 °C, placed in Petri-dishes (50 mm diameter) and moistened with 1 ml of sterile water. Groups of 12 workers were transferred to the Petri-dishes and video recorded behaviors were scored as described above. Survivorship was monitored for 2 weeks confirming that the treatment did not cause mortality. The experiment was replicated with workers from a separate colony to calculate an average alarm response (LOM) for each fungal isolate.

### TLC

Heptane or methanol extracts were spotted (10–25 µl) on TLC plates (Silica gel aluminum foils, Lichen Cottage, 10 × 10 cm) and sterols were separated with CHCl_3_:CH_3_OH: H_2_O mobile

phase (130:16:1, v: v:v) for 10 min and air dried (Yakobov et al. 2022). Plates were stained with a sulfuric acid/MnCl_2_ solution (concentrated sulfuric acid 9 ml, MnCl_2_.4H_2_O 0.8 g, CH_3_OH

120 ml, H_2_O 120 ml) and heated at 100 °C for approximately 15 min until the sterols were clearly visible.

### Termicin antifungal activity

Recombinant *R. flavipes* termicin was expressed in yeast by MyBioSource Inc (San Diego, CA, USA). The tag for purification was removed and the 37 amino acid sequence corresponded with the full length mature peptide: ACDFSSCWAGCQADHSINFRRAYCDGQHCVCVFVTGG.

Approximately 50 *Metarhizium* conidia were incubated for 24 h at 25 °C in a five µM solution of the peptide, or water for controls, in a total volume of 20 µl. There were 8 or 14 replicates of both treatment and control for 13 *Metarhizium* isolates. The conidial suspensions were plated on Petri-dishes (90 mm x 15 mm) with potato dextrose agar supplemented with 50 µg/ml ampicillin and incubated for 6–7 days at 25 °C. The antifungal activity was measured as the average percent reduction of colonies (colony forming units or CFUs) with termicin treatment relative to the controls.

Conidia (10 µl of 10^8^ conidia ml^-1^) from two isolates representing the least and most susceptible isolates to termicin antifungal activity were incubated with termicin (10 µl of 10 µM solution) or sterile water for controls (10 µl) for 10 min at 25 °C before dilution to 1 ml with sterile water. Groups of 12 workers were then exposed to 500 µl of the diluted termicin treated or untreated conidia (10^6^ conidia ml^-1^) or water for controls by brief immersion and agitation for ten seconds and monitored for survivorship as described above.

### Conidia adhesion

The strength of adhesion of conidia to the insect cuticle was compared for isolates MD002 and MD353, which represent minimum and maximum values in our correlational analyses between ergosterol content and in vivo LOMs. Conidia, 10^7^ suspended in 1 ml 0.01% Tween 80, were centrifuged at 8000 G for 2 min. The supernatant was discarded and the conidia were resuspended in sterile water. Conidia were counted with a hemocytometer and adjusted to a concentration of 5 × 10^6^ conidia ml^− 1^. Two replicates of 6 workers for each isolate were exposed to 250 µl of the 5 × 10^6^ ml^− 1^ conidia suspension. After immersion and gentle shaking for 10 s, the termites were deposited on filter paper to absorb excess liquid and set aside as single termites for 30 min in a 24 well plate, each well lined with moistened filter paper. The 12 workers were then combined and washed with 250 µl 0.1% Triton X-100 with gentle shaking for 20 s (modified from Ment et al. 2010). Two hundred µl of the wash was removed and centrifuged at 8000 G for 2 min. The supernatant was removed and the pellet was resuspended in 10 µl 0.1% Tween 80 for counting with a hemocytometer.

### Genetic distance

Concatenated EFα1, IGS and ITS DNA sequences were aligned with ClustalW (Thompson et al. 2003). The concatenated sequence was used to construct a phylogenetic tree with MrBayes using a general time reversible substitution model with invariable sites and gamma rate variation (Ronquist et al. 2012). The analysis was run for 10^5^ generations after a burn-in of 10^3^ generations. Pairwise genetic distances between the isolates were calculated with the concatenated sequence using the Jin-Nei correction method (Jin and Nei 1990). A Mantel test was used to test for a correlation between genetic distances between *Metarhizium* isolates (*n* = 12) and differences in ergosterol content or LOMs in response to conidia between the *Metarhizium* isolates or LOMs in response to ergosterol extracts from conidia. The Mantel test was also used for partial correlations of genetic distances between *Metarhizium* isolates and differences in ergosterol content and LOMs when controlling for species identity (same species or different species) or the latitude of the collection sites (same latitude or different latitude). TFS3 was considered as belonging to the same latitude as the other MD isolates collected at 39.4° N when controlling for latitude (same or different) in the Mantel tests.

### Statistics

Ergosterol content was measured by calculating total ergosterol plus 24(28)dehydroergosterol (24(28)DHE, a late sterol pathway intermediate), which has an absorbance peak at 281.5 nm, and then subtracting the amount due to 24(28)DHE only, which has an absorbance peak at 230 nm.

(Arthington-Skaggs et al. 1999). We used the following equation to calculate ergosterol content as a percentage of the conidia wet weight: [(A281.5/290)/pellet weight]- [(A230/518)/pellet weight], where 290 and 518 are the E values (in percentages per centimeter) determined for crystalline ergosterol and 24(28)DHE, respectively.

The average ergosterol content from two separate extractions from 13 isolates of *Metarhizium* were used to calculate correlation coefficients (Spearman rank, 2-tailed, alpha = 0.05). We used Cox regression so that we could compare the fatalities of termites challenged with the conidia of different isolates to untreated controls (hazard ratios of death) in a model encompassing all 13 isolates (Cox 1972). Cox regression was also used to calculate the hazard ratios of death (HRs) of termites exposed to conidia that had been either treated with termicin (10 min incubation of conidia with the antifungal peptide) or not treated with the peptide. For the preference test, an index of preference was calculated for eight replicates for each treatment: (difference between mean numbers of termites on treated and control filter paper) / (sum of means of termites on treated and control filter paper). A Kruskal Wallace Test was used for comparing preference indices across the three treatments, with follow up Dunns’ tests with a Bonferroni correction for pairwise comparisons. ANOVA was used for comparing means among treatments for LOMs and allogrooming in response to the treated filter papers, with follow up pairwise tests using Tukey’s HSD. For the comparison between estimates of the number of conidia that had attached to the cuticle for *Metarhizium* isolates MD002 and MD353, which represent minimum and maximum values in our correlational analyses between ergosterol content and in vivo LOMs, the mean number of conidia in 64 square areas of a hemocytometer measuring 0.25 mm^2^ (depth of 0.1 mm) was compared with a T-test (2-tailed, alpha = 0.05). We used IBM SPSS Statistics (version 28) for the majority of the statistical tests. The Mantel tests were performed with the program zt using 10^6^ randomizations (Bonnet and Van de Peer 2002).

## Results

### Preference tests for ergosterol

**Fig. 1:**
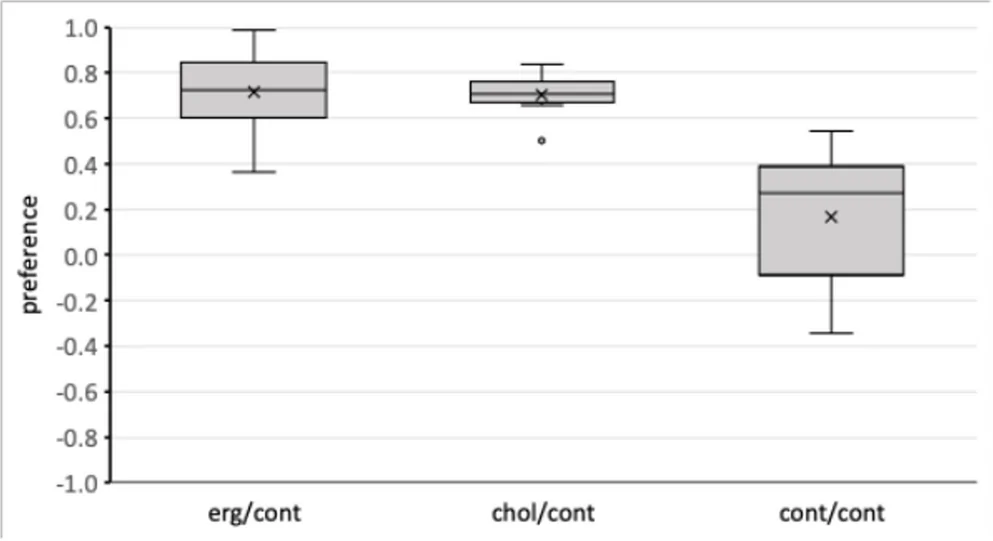
Preference of termites for treated circular filter paper halves: ‘erg’ corresponds with ergosterol treatment, ‘chol’ with cholesterol treatment, ‘cont’ (control) with solvent (ethanol) treatment alone. The x in the box plots corresponds with the mean, the box shows interquartile range around the median, the whiskers correspond with minimum and maximum values and the dot represents an outlier

**Fig. 2:**
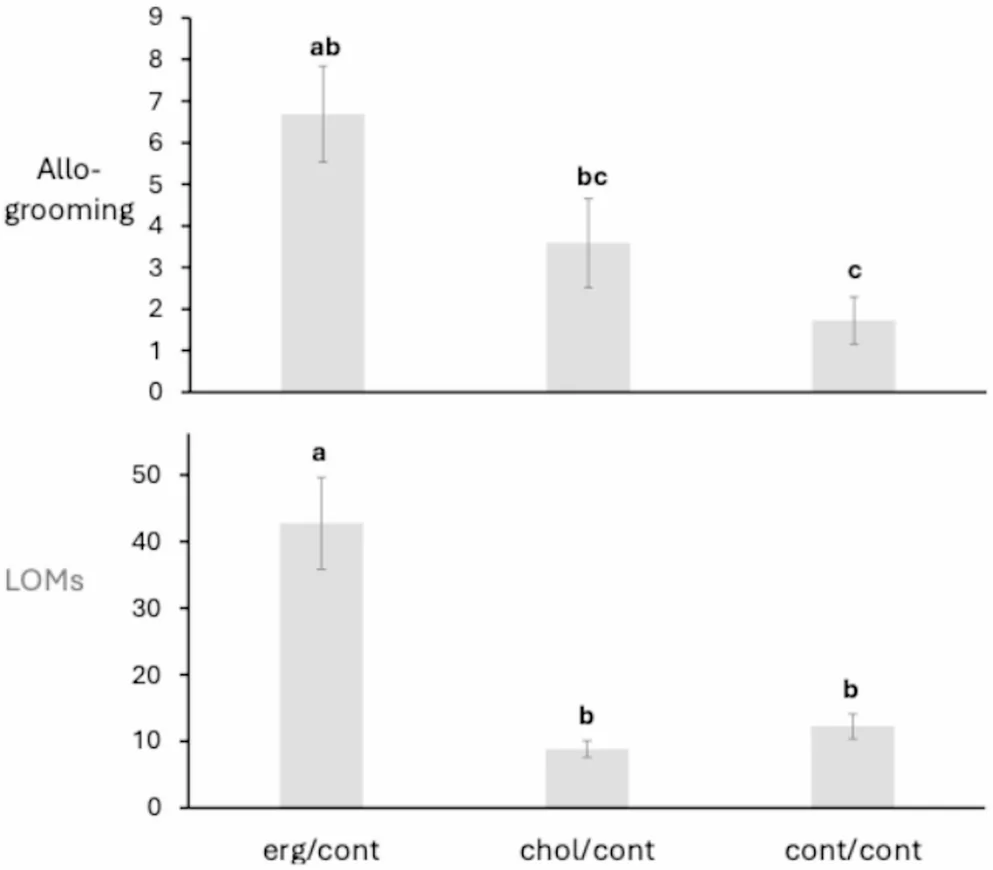
Termite LOMs and allogrooming in response to the filter paper halves: ‘erg’ corresponds with ergosterol treatment, ‘chol’ with cholesterol treatment, ‘cont’ (control) with solvent (ethanol) treatment alone. The error bars correspond with the standard deviation. Different letters indicate significant differences

Cholesterol and ergosterol significantly impacted how long termites spent on treated filter paper halves compared to the solvent controls. A Kruskal Wallace Test indicated significant differences among the group means (Chi-square = 13.5, *p* = 0.001). Dunns’ tests with a Bonferroni correction identified significant differences between occupancy of one side for cholesterol/control and ergosterol/control compared to the control/control (*p* = 0.002 and *p* = 0.001, respectively), with termites appearing to aggregate when occupying the sterol treatment side. There were no significant differences between ergosterol/control and cholesterol/control (*p* = 0.9436, Fig. 1). There were significant differences in the average LOMs among treatments (F = 19.97, df = 2, *p* < 0.001). Tukey’s HSD tests indicated significant differences in LOMs between the ergosterol/control treatment (mean LOMs = 42.75) and cholesterol/control (mean LOMs = 8.88), as well as between ergosterol/control and control/control (mean LOMs = 12.25). The comparison between cholesterol/control and control/control was not significantly different (Fig. 2). For allogrooming there was a significant difference across treatments, (F = 6.07, df = 2, *p* = 0.008). Tukey’s HSD indicated significant differences between ergosterol/control and control/control (mean allogrooming bouts = 6.13 and 1.38 respectively). The comparison between cholesterol/control (mean allogrooming bouts = 3.50) and the control/control was not significantly different (Fig. 2).

### Survival analysis

**Fig. 3:**
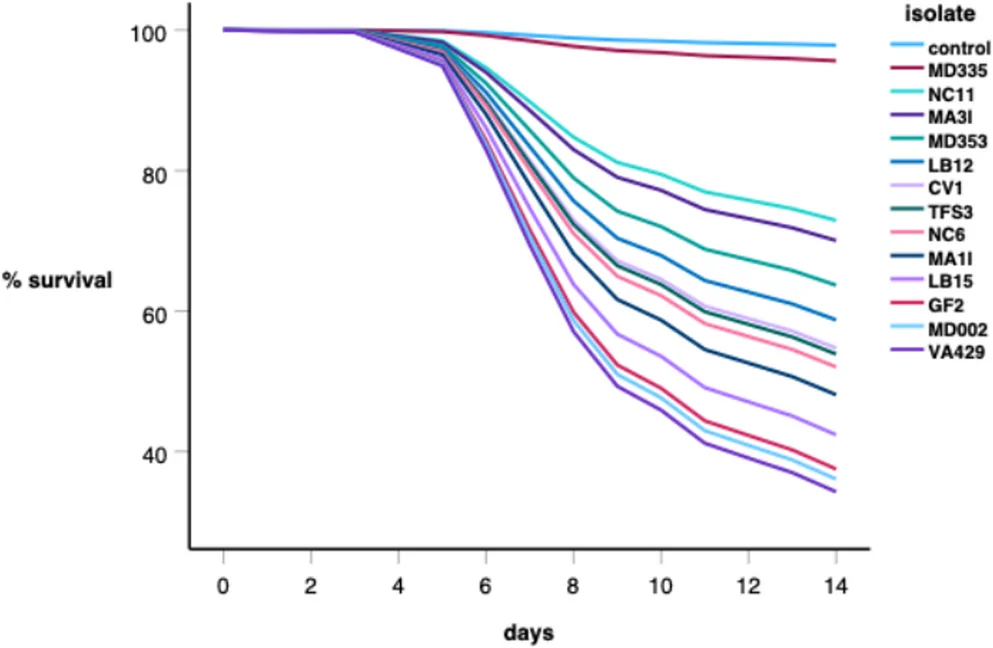
Cox regression of fatal infections. Groups of 12 termites (3–4 replicates) were challenged with 10^6^ conidia ml^− 1^ for each isolate. The order of the legend from top to bottom corresponds with the order of the curves. The cox regression statistics for each curve are provided in Table 1

The overall Cox regression model of survival was significant (Wald = 50.08, df = 13, *p* < 0.001). Termites treated with all the isolates except one showed significant hazard ratios of death relative to the controls (Table 1; Fig. 3).

**Table 1:** Cox regression statistics for survival analysis are shown in the left side of the table including results for the overall model. For species identification (spp.), Mb corresponds with *M. brunneum*, Mg with *M. guizhouense*, Mr with *M. robertsii*. Significance (sig.) indicates whether the hazard ratio of death (HR) was significantly different from controls (4 replicates of 12 termites that were not treated with conidia). At least two *R. flavipes* colonies were used for the replicates for each isolate and the controls. For each isolate, the estimate of ergosterol content [erg], which was an average of two replicates, and termicin antifungal activity were calculated as described in the Methods. Termicin antifungal activity represents the percent reduction of CFUs after conidia incubation with termicin relative to controls. An asterisk for termicin antifungal activity indicates that the termicin treatment was significantly different from controls (independent samples t-test, 2-tailed, alpha = 0.05). For the behavioral responses to conidia, cLOM corresponds with the number of bouts of LOMs after termites were challenged with conidia averaged over the number of replicates (rep.) and mLOM corresponds with the number of bouts of LOMs after termites were exposed to filter paper treated with methanol extracted ergosterol, averaged over 2 replicates. The latitude coordinates (lat.) are in degrees North (WGS84 / EPSG:4326)

Isolate	spp.	slope		SE	Wald	df	sig.	HR	rep.	[erg]	termicin activity sig.*	cLOM	mLOM	lat.
model					50.08	13	< 0.001							
MD002	Mb	3.84		1.02	14.29	1	< 0.001	46.60	4	0.514	86.01*	36.75	110.5	39.4
MD353	Mb	3.03		1.03	8.65	1	0.003	20.63	4	-0.132	2.91	93.50	36.0	39.4
VA429	Mb	3.89		1.02	14.68	1	< 0.001	48.95	4	0.185	73.14*	72.50	118.0	38.8
CV1	Mb	3.32		1.04	10.12	1	0.001	27.55	3	0.235	46.49*	36.89	92.0	39.4
TFS3	Mb	3.34		1.04	10.28	1	0.001	28.27	3	0.176	39.02	21.56	52.5	39.6
MD335	Mg	0.72		1.41	0.26	1	0.612	2.05	4	0.113	-12.25	165.30	86.0	39.4
NC11	Mg	2.67		1.04	6.62	1	0.01	14.45	4	-0.013	15.00	85.25	45.0	35.8
GF2	Mr	3.80		1.03	13.66	1	< 0.001	44.82	3	0.335	34.76*	27.22	82.0	39.4
LB12	Mr	3.19		1.03	9.68	1	0.002	24.32	4	0.054	-8.93	125.00	71.0	30.7
LB15	Mr	3.67		1.02	13.00	1	< 0.001	39.24	4	0.238	72.39*	61.00	120.0	30.7
MA1I	Mr	3.51		1.03	11.52	1	< 0.001	33.47	3	0.051	59.62*	23.00	90.5	42.4
MA3I	Mr	2.79		1.06	6.90	1	0.009	16.28	3	0.113	3.10	32.22	60.5	42.4
NC6	Mr	3.40		1.02	11.07	1	< 0.001	29.85	3	0.141	79.52*	77.50	77.0	35.8

### Ergosterol and LOMs

**Fig. 4:**
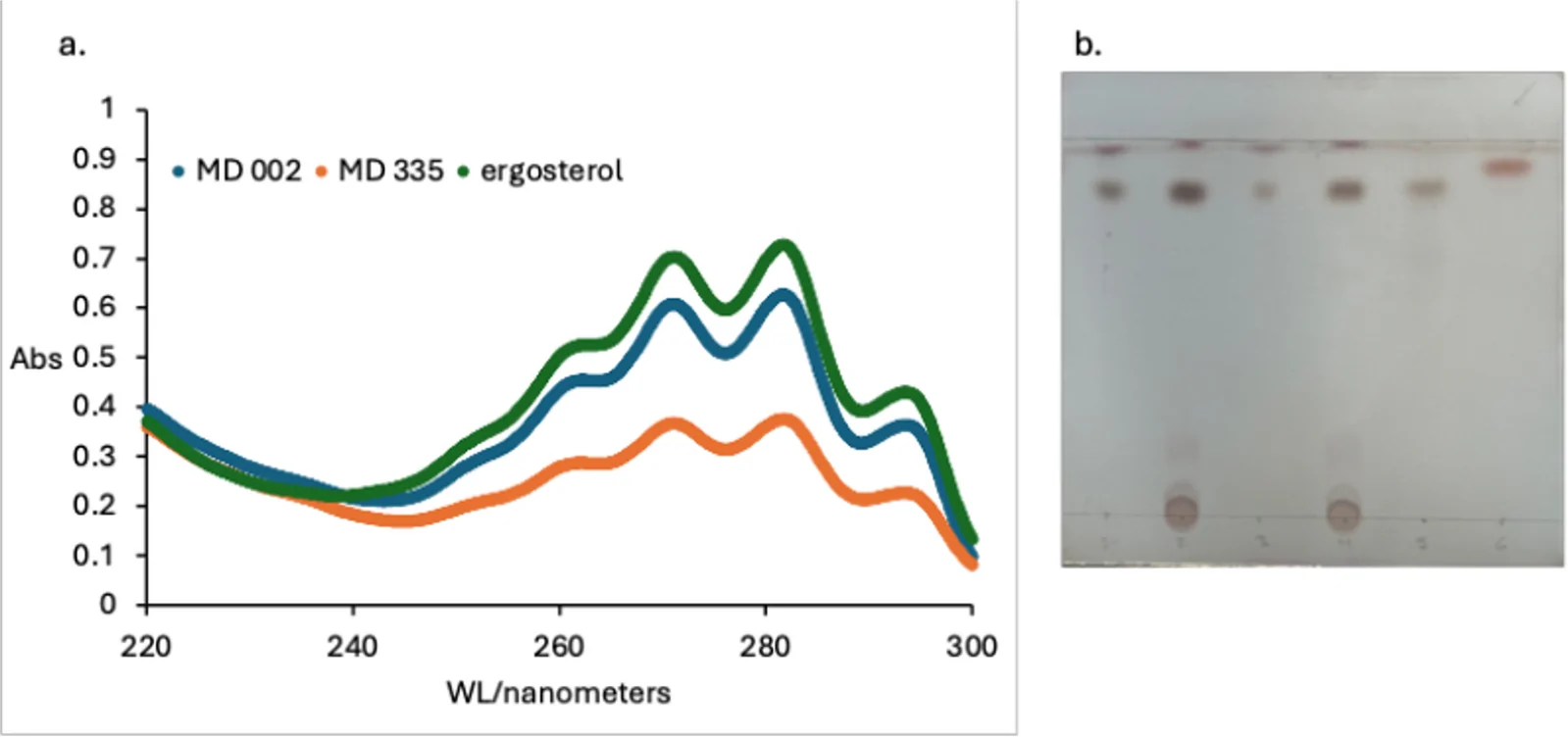
(a) Absorbance profiles of saponified ergosterol extracted from an isolate of *M. brunneum* (MD 002) and *M. guizhouense* (MD 335) and spectrophometric grade ergosterol (300 µg/ml), (b) TLC analysis: lanes 1 and 2, saponified and non-saponified MD 002 respectively; Lanes 3 and 4, saponified and non-saponified MD 353 respectively; Lane 5, ergosterol control (3 µg); Lane 6, lanosterol control (3 µg)

**Fig. 5:**
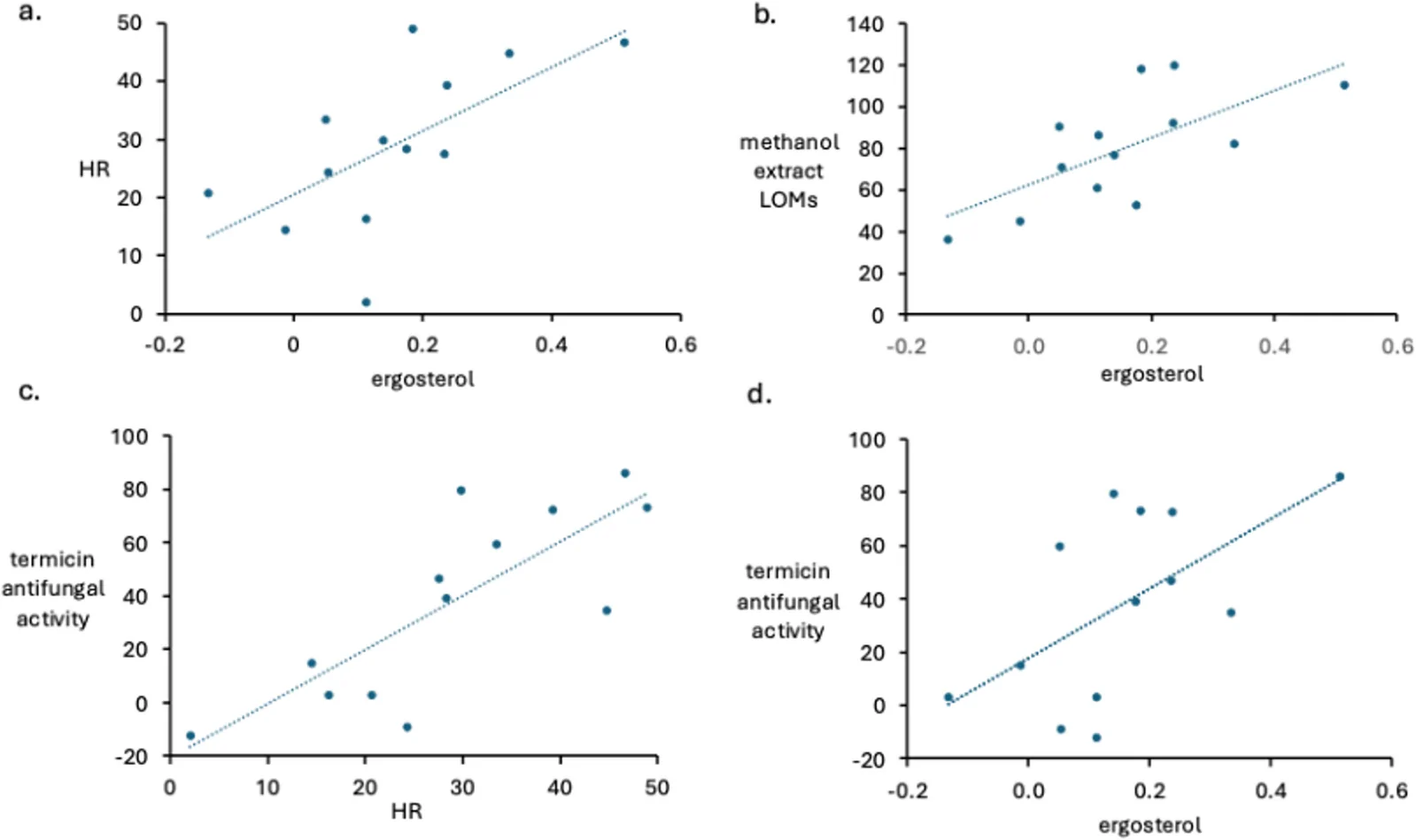
(a) the correlation (*r*_*s*_ = 0.692, *p* = 0.009) between the hazard ratio of death (HR) of groups of 12 termites challenged with the conidia of 13 different isolates of *Metarhizium* and estimates of saponified ergosterol content (% wet conidia weight) in each isolate, (b) The correlation (*r*_*s*_ = 0.698, *p* = 0.008) between the LOMs in response to filter paper treated with methanol extracts of ergosterol from conidia of 13 different isolates of *Metarhizium* and estimates of saponified ergosterol content (% wet conidia weight) in each isolate. The methanol (no ergosterol) and ergosterol control (300 µg) elicited 34 and 112.5 LOMs, respectively. (c) The correlation (*r*_*s*_ = 0.808, *p* < 0.001) between termicin fungicidal activity against the conidia of 13 different isolates of *Metarhizium* and the hazard ratio of death (HR) of groups of 12 termites challenged with conidia of the isolates. (d) The correlation (*r*_*s*_ = 0.588, *p* = 0.035) between termicin fungicidal activity against the conidia of 13 different isolates of *Metarhizium* and estimates of ergosterol content (% wet conidia weight) in each isolate

The saponified ergosterol extract spectrometer scans were almost identical to spectrophotometric grade ergosterol (Fig. 4a). Estimates of saponified ergosterol content (% conidia weight) were positively correlated with the hazard ratios of death (*r*_*s*_ = 0.692, *p* = 0.009, Fig. 5a). Estimates of methanol extracted ergosterol were not significantly correlated with the hazard ratios of death (*r*_*s*_ = 0.445, *p* = 0.128). The calculation of ergosterol content from methanol extractions appeared to be confounded by lipids or other compounds absorbing at 230 nm, which frequently resulted in negative values for estimates of total ergosterol. We therefore used absorbances of saponified sterols for measuring ergosterol content in subsequent analyses. LOMs in response to filter paper treated with methanol extracts of ergosterol were positively correlated with estimates of saponified ergosterol content (*r*_*s*_ = 0.698, *p* = 0.008, Fig. 5b). LOMs from challenges with conidia were not positvely correlated with estimates of saponified ergosterol content (*r*_*s*_ = -0.401, *p* = 0.174), which does not support the hypothesis that ergosterol is a recognition cue that elicits an alarm response to conidia detected on the termite cuticle. Moreover, there appeared to be a weak inverse relationship between the two variables.

### Conidia adhesion

The adhesion assay indicated that there was not a substantive difference in conidia contaminating workers’ cuticles after brief immersion in suspended conidia: the mean number of conidia released with Triton X-100 washes from isolates MD 002 (mean = 4.46 in 0.25 mm^2^) and MD 353 (mean = 3.95 in 0.25 mm^2^), which represent an isolate that elicited negligible alarm (MD 002) and an isolate that elicited elevated alarm (MD 353), were not significantly different (*t =* 1.704, df = 254, *p* = 0.09).

### TLC

Ergosterol from isolates MD 002 and MD 353, which represent the extremes in our correlational analysis in both ergosterol content and in vivo LOMs, was also analyzed by TLC. The conidia of MD 002, which is an isolate of *M. brunneum*, elicits a negligible alarm and allogrooming response and the conidia of MD 353, which is an isolate of *M. robertsii*, elicits a robust behavioral response. Modification of ergosterol by peroxidation or amino acid (AA) acylation could influence the behavioral response to conidia. AA acylation or peroxidation would also inhibit the migration of ergosterol in TLC plates (Nowak et al. 2016; Yakobov et al. 2022). However, TLC indicated that ergosterol was not modified by peroxidation or amino acid acylation (Fig. 4b). The retention of control ergosterol corresponds with both saponified and non-saponified (methanol extracted) ergosterol.

### Termicin

Workers exposed to conidia treated with termicin (5 µM) for 10 min were significantly more likely to survive than workers exposed to untreated conidia (workers challenged with untreated conidia 3.19 times more likely to die over 14 days, Wald = 4.821, *p* = 0.028) for isolate MD 002 but not isolate MD 353 (workers challenged with untreated conidia were not significantly more likely to die, Wald = 0.908, *p* = 0.341). There was no mortality in workers exposed to sterile water alone. For the observed difference in direct antifungal activity of termicin against MD 002 and MD 353 conidia, MD 002 also showed significant activity relative to controls (mean CFUs = 2.86 and 20.43, respectively, *t* = 7.769, df = 26, *p* < 0.001) but MD 353 did not (mean CFUs = 7.14 and 7.36, respectively, *t* = 0.094, df = 26,  *p* = 0.926).

Termicin antifungal activity was positively correlated with the hazard ratio of death (*r*_*s*_ = 0.808, *p* < 0.001, Fig. 5c), a significant correlation that had been observed previously with a smaller sample size (Luitel et al. 2024). Termicin antifungal activity was positively correlated with estimates of saponified ergosterol content (*r*_*s*_ = 0.588, *p* = 0.035, Fig. 5d).

### Genetic distance and LOMs

**Fig. 6:**
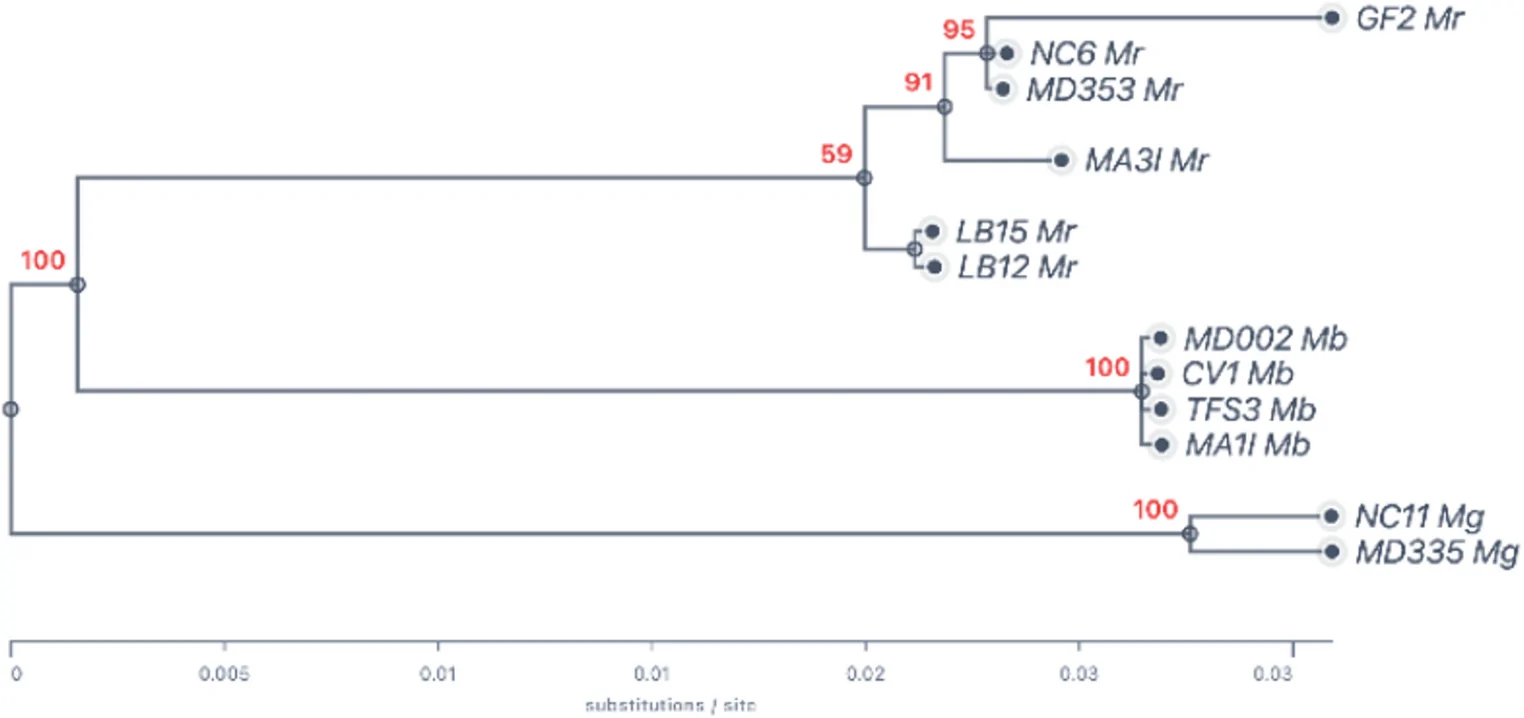
MrBayes tree of the *Metarhizium* isolates constructed with concatenated sequence (1508 nucleotides, GenBank accession numbers: EFα1, PZ842080-PZ842091; ITS, PZ437011-PZ437022; IGS, PZ446400-PZ446411). Mb corresponds with *M. brunneum*, Mg with *M. guizhouense*, Mr with *M. robertsii*. The credibility values (red) were 100 for the species lineage divergences. The analysis was run for 10^5^ generations after a burn-in of 10^3^ generations

Metarhizium isolates clustered according to species in the phylogenetic tree with credibility scores of 100 for each monophyletic grouping (Fig. 6). Genetic distances between the different *Metarhizium* isolates were significantly correlated with differences in the LOMs elicited by conidia (*r* = 0.332, *p* = 0.002), including while controlling for the latitude of the collection sites (*r* = 0.330, *p* = 0.003) but not species identity (*r* = -0.077, *p* = 0.270). Genetic distance between the different *Metarhizium* isolates were not significantly correlated with differences in LOMs elicited by extracted ergosterol (*r* = -0.098, *p* = 0.217), including while controlling the latitude of the collection sites (*r* = -0.084, *p* = 0.194) or species identity (*r* = -0.102, *p* = 0.211). Genetic distance between the different *Metarhizium* isolates were not significantly correlated with differences in saponified ergosterol content (*r* = − 0.056, *p* = 0.326), including while controlling for species identity (*r* = -0.090, *p* = 0.248) or the latitude of the collection sites (*r* = -0.077, *p* = 0.270).

## Discussion

Estimates of ergosterol conidial content (% wet weight) in different strains and species of *Metarhizium* were positively correlated with fatal infections, which does not support the hypothesis that ergosterol is the PAMP that elicits the protective effect of allogrooming (Bulmer et al. 2023). Ergosterol by itself did elicit elevated alarm and allogrooming, which has also been observed in *C. formosansus* (Chen et al. 2023). In the preference experiment, termites spent more time on the filter paper treated with either cholesterol or ergosterol, but only ergosterol elicited a significant increase in alarm and allogrooming. Moreover, the alarm induced with filter papers that had been treated with methanol extracted ergosterol was significantly positively correlated with the ergosterol content from different *Metarhizium* isolates. This elevated response to commercial or increasing concentrations of extracted ergosterol indicates that the molecule provides termites with a potential recognition cue, but not the initial cue that elicits the rapid allogrooming response to conidia contamination that limits fatalities. Cholesterol has been shown to be an endogenous component of an aggregation pheromone in *R. speratus* (Mitaka et al. 2020). Ergosterol also appears to be acting as an attractant or arrestant that facilitates *R. flavipes* worker aggregation and allogrooming.

In contrast to the significant positive correlation between the alarm response and the amount of extracted ergosterol, the intensity of the behavioral response to *Metarhizium* conidia was negatively correlated with ergosterol content. This correlation was not significant but does suggest that there is an inverse relationship between ergosterol and another PAMP that elicits alarm and allogrooming in response to conidia contaminating the cuticle. The integrity and make-up of the cell wall have been shown to be influenced by ergosterol content in fungi (Mesa-Arango et al. 2016; Ribeiro et al. 2022; Choy et al. 2023), which supports the hypothesis that ergosterol influences a cell wall PAMP that triggers the direct behavioral response to conidia. The cell wall presumably masks cell membrane molecules from direct detection which aligns with an indirect role for ergosterol influencing a cell wall PAMP that triggers the rapid hygienic response.

The antifungal activity of termicin was positively correlated with ergosterol content. Moreover, brief treatment of conidia with termicin was only effective at improving survival in challenged termites for the isolate with the highest ergosterol content (MD 002) and not for the isolate with the lowest ergosterol content (MD 353). The mechanism of termicin activity is unknown, although the amphipathic peptide is believed to disrupt the cell membrane (Lamberty et al. 2001; Koehbach 2017). This activity may release membrane bound ergosterol, which then produces a PAMP that can elicit a behavioral response, which includes aggregation, alarm and allogrooming. The release of ergosterol could therefore help counteract the weak alarm response to isolates with lower content of the PAMP that elicits initial allogrooming, because the two are inversely correlated. Chen et al. (2023) found residual ergosterol on the cuticles of *C. formosansus* workers that had been exposed to *Metarhizium* conidia for an hour, which may have been released by antifungal effectors such as termicin. The release of ergosterol could weaken the negative correlation between ergosterol and the LOMs elicited by conidia. Ergosterol may therefore become an important secondary elicitor of aggregation and allogrooming when the initial emergency alert elicited by an unidentified cell wall PAMP is weak or absent. Cell wall β-glucans, which would be excluded from the solvent in our extracts, are possible candidates for the cell wall PAMP that elicits allogrooming as they have been shown to activate the insect innate immune system after infection (Takahashi et al. 2015). However, inhibition of *R. flavipes* β-1,3-glucanases, which could help generate soluble β-glucan PAMPs, did not reduce allogrooming rates in termites that had been challenged with *Metarhizium* conidia (Esparza-Mora et al. 2020). Variation in conidia adhesion to the cuticle also did not appear to be responsible for differences in LOMs as there was no significant difference in our adhesion assay between an isolate that elicited negligible alarm (MD 002) and an isolate that elicited elevated alarm (MD 353).

The adaptive evolution of termicin (Bulmer et al. 2010) could be directed by *Metarhizium* isolates with high ergosterol content because these isolates are especially virulent, which is attributable to their conidia being difficult to initially detect. Evidence of selective pressure on termicin indicates that adaptive adjustments of the peptide are a response to changes in its target (Bulmer et al. 2010), which could be within or across different isolates. Ergosterol can be modified by peroxidation and amino acid acylation in *Metarhizium* and other fungi (Kobori et al. 2007; Yakobov et al. 2022). Since termicin is most effective against *Metarhizium* isolates that are not easily detected by workers, there could be an inverse relationship between ergosterol and a modified ergosterol that influences recognition of conidia during allogrooming. Although this might require release of modified ergosterol from the cell membrane, so it is not masked by the cell wall. The TLC analysis of MD 002 and MD 353, which represent the extremes in our correlational analysis in both ergosterol content and in vivo LOMs, did not reveal any modifications of the sterol that would shift its position or produce additional bands corresponding with modified ergosterol. Termicin activity may depend on additional lipids that are directly influenced by ergosterol content. Amphipathic bee venom peptides are also more effective against yeast with higher ergosterol content but do not interact directly with the sterol (Kodedová et al. 2019). Lipid rafts composed of sphingolipids and ergosterol appeared to be important for the interaction between the bee peptides and yeast cells. Lipid rafts also influence the composition of the cell wall (Mollinedo 2012).

Our results suggest an evolutionary and mechanistic inter-play between termicin activity and hygienic behavior. In this mechanistic model, the behavioral response compensates for weaker termicin fungicidal activity against isolates with lower cell membrane ergosterol. As a result of a counter balanced interaction between the cell membrane and cell wall, isolates with lower ergosterol content have higher content of the PAMP that elicits alarm and allogrooming. This behavioral response is likely to be energetically costly. However, it can be fine-tuned by selection minimizing the cost of fatal infections because there is a compensatory balance between the detectability of conidia and the damage that termicin can inflict on these pathogenic propagules. Furthermore, in isolates with low content of the PAMP that elicits the initial hygienic response, termicin activity could help compensate for this vulnerability by releasing membrane bound ergosterol, which acts as a secondary PAMP that increases allogrooming.

It remains unclear if selective change in termicin reflects a counter response to changes made to the fungal cell membrane to evade the weapon or reflects a response to termites frequently encountering novel virulent isolates, which have elevated ergosterol content for other adaptive or fortuitous physiological reasons. The significant correlation between differences in *Metarhizium* isolate genetic distance and differences in LOMs elicited by their conidia indicates that the PAMP that elicits the behavioral response has diverged genetically with the diversification of the fungus. In contrast, the lack of correlation between differences in genetic distance and ergosterol content (with or without controlling for species identity or latitude), or the LOMs elicited by extracted ergosterol, suggests that ergosterol content and the direct behavioral response to the sterol are not influenced by genetic relatedness. Instead, ergosterol content appears to be a more plastic trait that may be influenced by environmental conditions. Serial exposure of *Metarhizium* spp. to small groups of Argentine ants in the lab increased the pathogen’s ability to evade the ants’ hygienic response, apparently as a result of a reduction in ergosterol content (Stock et al. 2023). This plasticity indicates that ergosterol can be quickly reduced by local strains of *Metarhizium*, decreasing their susceptibility to termicin so that they can more effectively exploit dense populations of patchily distributed termites. However, the tradeoff for reducing cell membrane ergosterol is an increase in a cell wall PAMP that termites can easily recognize.

## Data Availability

Data available in a publicly accessible repository. The data presented in this study are openly available in Zenodo at: https://doi.org/10.5281/zenodo.20287310.
